# Relationship of ethnicity and CD4 Count with glucose metabolism among HIV patients on Highly-Active Antiretroviral Therapy (HAART)

**DOI:** 10.1186/1472-6823-13-13

**Published:** 2013-04-22

**Authors:** Ranjita Misra, Prakash Chandra, Steven E Riechman, Dustin M Long, Shivani Shinde, Henry J Pownall, Ivonne Coraza, Dorothy E Lewis, Rajagopal V Sekhar, Ashok Balasubramanyam

**Affiliations:** 1 Department of Social and Behavioral Sciences, School of Public Health, West Virginia University, Morgantown, USA; 2 Translational Metabolism Unit, Division of Diabetes, Endocrinology and Metabolism, Baylor College of Medicine, Houston, TX, USA; 3 Department of Biostatistics, West Virginia University, Morgantown, USA; 4 Section of Atherosclerosis and Lipoprotein Research, Baylor College of Medicine, Houston, TX, USA; 5 University of Texas School of Public Health, Houston, TX, USA; 6 Legacy Community Health Services, Houston, TX, USA; 7 Department of Internal Medicine, University of Texas Medical Branch, Galveston, TX, USA; 8 Endocrine Service, Ben Taub General Hospital, Houston, TX, USA

**Keywords:** African American, Hispanic, Impaired glucose tolerance, HbA1c, Dyslipidemia

## Abstract

**Background:**

HIV patients on HAART are prone to metabolic abnormalities, including insulin resistance, lipodystrophy and diabetes. This study purports to investigate the relationship of ethnicity and CD4+ T cell count attained after stable highly-active antiretroviral treatment (HAART) with glucose metabolism in hyperrtriglyceridemic HIV patients without a history of diabetes.

**Methods:**

Demographic, anthropometric, clinical, endocrinologic, energy expenditure and metabolic measures were obtained in 199 multiethnic, healthy but hypertriglyceridemic HIV-infected patients [46% Hispanic, 17% African-American, 37% Non-Hispanic White (NHW)] on stable HAART without a history of diabetes. The relationship of glucose and insulin responses to ethnicity, CD4 strata (low (<300/cc) or moderate-to-high (≥ 300/cc)), and their interaction was determined.

**Results:**

African-Americans had significantly greater impairment of glucose tolerance (P < 0.05) and HbA1c levels (P < .001) than either Hispanics or NHWs. In multivariate models, after adjusting for confounders (age, sex, HIV/HAART duration, smoking, obesity, glucose, insulin and lipids), African-Americans and Hispanics had significantly higher HbA1c and 2-hour glucose levels than NHW’s. Demonstrating a significant interaction between ethnicity and CD4 count (P = 0.023), African Americans with CD4 <300/cc and Hispanics with CD4 ≥300/cc had the most impaired glucose response following oral glucose challenge.

**Conclusions:**

Among hypertriglyceridemic HIV patients on HAART, African-Americans and Hispanics are at increased risk of developing diabetes. Ethnicity also interacts with CD4+ T cell count attained on stable HAART to affect post-challenge glycemic response.

## Background

HIV/AIDS affects U.S. minorities disproportionately, reflecting a change in the distribution of the disease by racial/ethnic groups since the beginning of the epidemic [1,2]. The widespread use of highly-active antiretroviral therapy (HAART) has slowed the progression of HIV infection to AIDS and has reduced associated morbidity and mortality [3]. However, HIV patients on HAART are prone to metabolic abnormalities, including insulin resistance, lipodystrophy and diabetes (1). Lipodystrophy - characterized by peripheral fat loss, visceral fat increase, hypertriglyceridemia, and low HDL-C - is often associated with insulin resistance [4,5], a risk factor for developing diabetes. Although the frequencies of impaired glucose tolerance (IGT) and diabetes have not been systematically evaluated in HIV patients of different ethnicities and geographic areas, the rates are likely to be higher than in the general population. An early study of HIV patients with lipodystrophy and abdominal fat accumulation reported a 35% prevalence of IGT and 8% prevalence of diabetes, compared to 5% and 0.5% respectively for matched non-HIV controls [4]. The high frequency of obesity (an independent risk factor for metabolic abnormalities and CVD) among HIV/HAART patients has an inimical effect on their immune reconstitution and body composition [6].

Diabetes and cardiometabolic syndrome are highly prevalent among ethnic minorities in non-HIV infected populations [7]. The relationship between HbA1c and fasting serum glucose is not homogenous across racial/ethnic groups: e.g., African-Americans and Hispanics have higher HbA1c than non-Hispanic Whites (NHWs) after adjusting for fasting glucose concentration, glucose area under the curve after oral glucose, insulin response and insulin resistance [8,9]. Possible important interactions between ethnicity and specific aspects of glucose metabolism may be obscured in epidemiologic studies that utilize limited parameters (e.g., only fasting serum glucose, or HbA1c) to measure dysglycemia: e.g., significant differences have been noted in HbA1c levels between African-Americans and NHWs at elevated serum glucose levels [8]. Among HIV patients, elevated HbA1c has been associated with higher CD4 cell count, older age, and ethnicity [10]. Similar data from relatively small studies and heterogeneous HIV populations suggest that metabolic (including glycemic) abnormalities among HIV patients may also be affected by ethnicity [11,12]. However, in the context of diabetes risk, the relationship of ethnicity and its interactions with metabolic parameters and the degree of immune reconstitution following HAART have not been investigated systematically in HIV patients.

The purpose of this study was to assess the relationship of ethnicity with several simultaneously measured glycemic parameters - HbA1c, fasting serum glucose and insulin, and post-challenge serum glucose and insulin levels - among hypertriglyceridemic HIV patients on stable HAART who participated in the Heart Positive study. We hypothesized that ethnicity and CD4 level on stable HAART would exert an influence on glycemic regulation in these hypertriglyceridemic but otherwise healthy HIV patients.

## Methods

Heart Positive (Clinicaltrials.gov ID NCT00246376) was a large multiethnic study of healthy patients with HIV infection on stable HAART [13]. The primary outcomes have been reported [13]. The protocol was approved by the Institutional Review Boards of Baylor College of Medicine and the two recruitment centers. Written informed consent was obtained (in Spanish or English) from all subjects prior to their participation.

### Subjects

HIV patients with no history of diabetes were recruited from two HIV centers in Houston: Legacy Community Health Services and Thomas Street Clinic, Harris County Hospital District. The sample comprised 202 patients on stable HAART with hypertriglyceridemia who underwent complete screening for the Heart Positive study. Racial/ethnic distribution of the subjects included NHWs, African Americans, Hispanics, and Native Americans. Three Native American subjects were excluded from this analysis due to their small numbers. All subjects were hypertriglyceridemic (fasting plasma triglycerides >150 mg/dL), on no lipid-lowering or anti-diabetic medications, on stable HAART for at least 6 months, with stable CD4 count and viral load, normal kidney and thyroid functions, and transaminase (AST / ALT) levels ≤ 2 times upper limit of normal.

### Measures

#### Demographic and clinical variables

Age, gender, ethnicity, number of years since HIV diagnosis, protease inhibitor (PI) use, smoking, alcohol use, drug abuse, and duration of HAART.

#### Body composition

Was measured by bioelectrical impedance analysis (Quantum II Analyzer, RJL Systems, Clinton Township, MI). Body Mass Index and waist and hip circumference were measured.

#### Glucose and insulin

Subjects ingested 75 g glucose (SunDex, Fisher Health Care, Houston, TX) in the morning after a 10 h overnight fast, with blood sampling at 0 (fasting), 30, 60, 90 and 120 minutes. Plasma glucose was measured by the glucose oxidase method and insulin by radioimmunoassay (Linco, St. Louis, MO). HbA1c was measured by HPLC at The Methodist Hospital clinical laboratory, Houston, TX.

#### CVD risk markers

Total cholesterol, HDL-C and triglyceride profiles were determined by an automated enzymatic method (Olympus AU400e Analyzer, Center Valley, PA) at the Baylor Atherosclerosis Core Laboratory (CLIA-CAP certified). LDL-C was calculated using the Friedewald formula. Fasting plasma total cholesterol, HDL-C and triglycerides were measured in the Baylor Atherosclerosis Clinical Laboratory using an Olympus AU400e automated analyzer. The median interassay c.v.’s for these assays were < 2–5%, and the median intra-assay c.v.’s were < 2–8%.

#### Energy expenditure

Resting energy expenditure (REE) was measured at the Baylor General Clinical Research Center (GCRC) by indirect calorimetry (Deltratrac, Sensormedics, Fullerton, CA) and calculated as REE = VO2 x [4.686 + (RQ-0.707) * 0.361/0.293 [14]. For subjects who could not visit the GCRC (n = 68), REE was measured using a MedGem device (Microlife USA, Dunedin, FL) that measures VO_2_ and calculates REE using a modified Weir equation.

#### CD4+ T cell count and HIV-1 viral load

CD4 count was measured by flow cytometry. To assess the relationship of CD4 levels to glucose abnormalities, subjects were divided into two groups: “low” (<300/cc; n = 53), and “moderate-high” (≥300/cc; n = 147). The cutoff of 300/cc was based on the World Health Organization’s current recommendation to the increase the threshold for the use of antiretroviral therapy (ART) for HIV-infected individuals to a CD4 count of ≤350 cells/cc (from the previous level of ≤200 cells/μL) [15]. In view of these changes that occurred during the period of the Heart Positive study, we used a cutoff slightly lower than that of the WHO to define the CD4 strata. HIV-1 viral load (VL) was measured by LabCorp or Quest Diagnostics (Madison, NJ) using either of two quantitative real-time PCR assays (routine, with a lower limit of 400 copies/cc, or ultrasensitive, with a lower limit of 50 copies/cc).

VL values measured as <400/cc by the first assay were assigned a value of 200/cc and all the VL values were log-transformed prior to analysis.

### Statistical analysis

Descriptive measures, chi-square (χ^2^) analysis and ANOVA were used to examine associations and differences in metabolic, hormonal, anthropometric and clinical parameters by ethnicity and CD4 strata. When differences in groups were observed, Bonferroni Post-Hoc analysis was performed to determine which groups were different from each other. Repeated measures ANOVA was used to assess the relationship of ethnicity with glucose measures following the oral challenge. Covariates were age, sex, BMI, lean mass, REE/lean mass, HAART duration, PI usage, HIV duration, fasting glucose level and fasting insulin level. Two-way interactions between ethnicity and glucose, ethnicity and gender, ethnicity and age, and ethnicity and CD4 count were included in the model and removed if non-significant (p > 0.05). Linear regression was performed with fasting serum glucose, 2-hour glucose, and HbA1c as outcome variables, and age, sex, BMI, waist, lean mass, REE/lean mass, HAART duration, PI usage, HIV duration, fasting glucose, HOMA-IR, systolic and diastolic blood pressure, family history of diabetes and interactions as independent variables. To avoid colinearity, BMI and lean mass (and not waist-hip-ratio and fat mass) were included in the final model. All statistical analyses were performed using the Statistical Package for Social Sciences (SPSS) (version 16 .0, SPSS Inc, Chicago).

## Results

Table 1 summarizes demographic, anthropometric and clinical parameters of the patients by ethnicity and CD4 strata. Mean age was 43.6 ± 8.3 years, duration since HIV diagnosis 9.5 ± 5.9 years, and duration of HAART 5.6 ± 4.4 years. One quarter of the subjects (26%) had CD4 <300 / ml. Ethnic distribution was 46% Hispanic, 17% African-American and 37% NHW. The majority of the patients were indigent and uninsured. Plasma HIV-1 viral load was undetectable in 74%. There was no difference in the frequency of patients with undetectable viral load with respect to ethnic group or CD4 stratum. Fourteen subjects had HIV/HCV co-infection. However, mean fasting glucose was similar between those with and without the co-infection [P = 0.204].

**Table 1:** Demographic, clinical, endocrine, HIV, metabolic, glycemic and insulin sensitivity parameters, by ethnicity and CD4 strata

**Variables**	**Total (n = 199)**	CD4 Count*	CD4 Count*	**F-Value**	**P**	**Ethnicity**	**Ethnicity**	**Ethnicity**	**F-Value/χ**^**2 **^**value**	** P**
		**Low (<300/cc; n = 53)**	**Moderate-High (> = 300; n = 146)**			**Non Hispanic White (n = 74)**	**African- American (n = 33)**	**Hispanic (n = 92)**		
**Demographic, Clinical, HIV**										
Age	43.6 ± 8.3	43.7 ± 7.6	43.5 ± 8.5	.026	.871	46.9 ± 8.1	45.9 ± 7.1	39.9 ± 7.4	19.449	<.001
HIV duration (y)	9.5 ± 5.9	9.9 ± 6.2	9.4 ± 5.9	.215	.643	12.7 ± 5.9	7.8 ± 5.1	7.4 ± 5.1	21.803	<.001
HAART duration (y)	5.6 ± 4.4	5.3 ± 5.1	5.7 ± 4.1	.417	.519	7.1 ± 5.2	5.1 ± 4.1	4.6 ± 3.5	6.955	.001
PI Drug Use	67.8%	61.0%	86.5%	11.46	.001	73.0%	66.7%	64.1%	1.49	.474
Family History of Diabetes	42.7%	43.1%	42.5%	.007	.933	28.4%	36.4%	57.1%	14.48	.001
Smoking	61.8%	65.4%	61.0%	.319	.572	75.7%	69.7%	47.8%	14.51	.001
Drug Use	28.1%	28.8%	28.1%	.011	.916	35.1%	36.4%	19.6%	6.24	.044
SBP (mm Hg)	128.3 ± 14.4	130.5 ± 15.7	127.5 ± 13.9	1.741	.188	131.5 ± 15.3	130.8 ± 14.9	125.5 ± 13.4	4.050	.019
DBP (mm Hg)	82.4 ± 10.3	83.4 ± 11.4	82 ± 9.9	.730	.394	84.3 ± 11.2	82.1 ± 10.9	81.2 ± 9.3	1.914	.150
Log Viral load	2.41 ± .40	2.41 ± .48	2.41 ± .38	.002	.961	2.36 ± .40	2.48 ± .08	2.41 ± .39	.920	.400
Total Chol. (mg/dL)	211.4 ± 48.5	207.6 ± 43.5	212.8 ± 50.2	.441	.507	218.1 ± 58.1	201.9 ± 42.0	208.3 ± 41.9	1.520	.221
TG (mg/dL)	302 ± 163	321 ± 145	295 ± 169	1.030	.311	323 ± 163	247 ± 118	302 ± 172	2.567	.079
HDL-C (mg/dL)	39.7 ± 8.9	38.1 ± 8.3	40.3 ± 9.2	2.461	.118	39.6 ± 9.5	39.2 ± 10.1	39.9 ± 8.4	.084	.920
Calc. LDL-C(mg/dL)	111.9 ± 40.4	105.3 ± 37.7	114.3 ± 41.2	1.927	.167	113.9 ± 43.7	113.3 ± 33.4	108.7 ± 41.2	.369	.692
**Metabolic**										
FFA (mmol/L )	.32 ± .22	.29 ± .17	.33 ± .24	1.21	.273	.34 ± .25	.30 ± .18	.32 ± .21	.33	.72
Leptin (ng/mL)	6.8 ± 5.9	5.5 ± 4.8	7.2 ± 6.3	3.193	.076	6.3 ± 5.3	8.1 ± 7.1	6.6 ± 5.9	1.02	.364
Adiponectin (μg/mL)	6.67 ± 5.39	7.40 ± 5.81	6.31 ± 5.16	1.00	.319	8.00 ± 6.46	4.90 ± 5.31	6.25 ± 4.0	2.78	.066
hsCRP (mg/L)	3.73 ± 5.48	3.51 ± 5.86	3.80 ± 5.33	.105	.747	3.48 ± 4.67	4.40 ± 5.45	3.67 ± 6.04	0.32	.73
Percent body fat (%)	22.1 ± 8.2	20.5 ± 6.3	22.7 ± 8.8	2.589	.109	19.5 ± 6.8	24.8 ± 8.1	23.3 ± 8.8	5.97	.003
BMI (kg/m^2^)	26.8 ± 3.5	26.3 ± 3.2	27 ± 3.6	1.735	.189	25.9 ± 3.6	27.7 ± 3.2	27.3 ± 3.5	4.43	.013
Waist circ. (cm)	91.5 ± 11.7	90.2 ± 14	91.9 ± 10.7	.799	.372	93.4 ± 11.3	93.4 ± 12.5	89.5 ± 11.8	2.47	.087
Waist-hip ratio	.9 ± .1	.9 ± .1	.9 ± .09	.141	.708	.96 ± .07	.98 ± .18	.93 ± .09	3.62	.029
VCO_2_ (mL/min)	221 ± 42.4	231.3 ± 44.3	216.8 ± 41	3.690	.057	235.3 ± 42.6	223.7 ± 47.6	204.9 ± 34.5	8.64	<.001
VO_2_ (mL/min)	265.3 ± 48.3	279.7 ± 48.4	259.8 ± 47.4	6.489	.012	283.5 ± 50.7	264.3 ± 48	249.2 ± 41	10.44	<.001
RQ	.83 ± .06	.83 ± .07	.84 ± .06	.066	.797	.84 ± .08	.84 ± .04	.84 ± .05	.008	.992
REE (kCal/24 h)	1819.1 ± 357	1897 ± 320	1789.8 ± 367	1.128	.290	1965.3 ± 357	1838.8 ± 344	1691.2 ± 320	13.05	<.001
REE/Lean Mass	121.0 ± 62.7	129.1 ± 58.2	117.8 ± 64.3	1.157	.284	142.5 ± 72.9	94.5 ± 38.2	113.1 ± 56.1	7.516	.001
**Glycemia, Insulin Sensitivity**										
Insulinogenic Index	.59 ± .4	.62 ± .4	.64 ± .4	.093	.761	.59 ± .5	.56 ± .3	.60 ± .4	1.064	.347
HOMA-IR	3.3 ± 3.9	3.5 ± 4.1	3.3 ± 3.9	.061	.805	3.3 ± 3.7	2.9 ± 2.3	3.5 ± 4.7	.329	.720
Fasting glucose (mg/dl)	94.7 ± 14.2	95 ± 14.03	94.6 ± 14.4	.037	.848	93.1 ± 8.6	97.8 ± 22.8	94.8 ± 14	1.266	.284
OGTT Glucose 120 min (mg/dl)	127.9 ± 41.7	124.7 ± 47.7	129.1 ± 39.4	.436	.510	121.6 ± 37.6	143.4 ± 51.4	127.1 ± 40.3	3.126	.046
Fasting Insulin (μU/ml)	15.4 ± 25.6	14.7 ± 18.6	15.6 ± 27.8	.048	.827	14.5 ± 17.2	20.1 ± 49.7	14.5 ± 17.8	.653	.522
OGTT Insulin 120 min (μU/ml)	83.7 ± 76	75.1 ± 74.3	86.8 ± 76.7	.903	.343	63.3 ± 56.9	81.4 ± 58	101.9 ± 90.9	5.424	.005
HbA1c (%)	5.3 ± .6	5.3 ± .6	5.3 ± .56	.174	.677	5.3 ± .45	5.69 ± .74	5.22 ± .57	8.207	<.001

Data are mean ± SE or percent.

Hispanics were significantly younger, had shorter HIV infection duration, fewer years of HAART and lower systolic blood pressure, and reported a lower rate of smoking, drug use and family history of diabetes, than African-Americans or NHWs (based on Bonferroni Post-Hoc analysis). Furthermore, they had significantly lower weight, height and body cell mass than African-Americans or NHWs. African-Americans had significantly more fat mass than Hispanics or NHWs (P < 0.01). Both African-Americans and Hispanics had higher BMI and percent body fat than NHW’s. NHWs had higher resting VCO_2_, VO_2_ and REE than Hispanics or African-Americans (P < 0.001). There were no significant ethnic diffenences in frequency of PI use or in fasting plasma levels of lipids, adiponectin, hsCRP, leptin, and free fatty acids. or.

No significant differences were noted for frequency of use of first generation PI drugs (indinavir, nelfinavir, saquinavir) compared to more recent PI drugs (atazanavir, darunavir), or for use of thymidine analogue drugs, with respect to ethnic group or CD4 stratum. No differences were noted between the low and moderate-high CD4 groups in demographic / clinical / anthropometric or endocrine / hormonal / energy expenditure parameters except for a higher frequency of PI use and lower body cell mass among subjects with moderate-high CD4 count, higher testosterone level in the low CD4 group, and higher VO_2_ with a trend towards higher VCO_2_ in the low CD4 group.

Insulin sensitivity and glycemic parameters by ethnicity and CD4 cell countare presented in Table 1. The 2-hour glucose value in the OGTT differed significantly by ethnicity but not by CD4 strata. IGT (defined by 2-h glucose level >140 but <200 mg/dl) was observed in 32% of the subjects - these were significantly older than those with normal glucose tolerance (P < 0.001). Compared to either Hispanics or NHWs, African-Americans manifested greater impairment of glucose tolerance, as indicated by higher 2-hour glucose (P < 0.05) and HbA1c (P < 0.001) levels. Hispanics had higher insulin levels at 90 min and 120 min of the OGTT than NHW’s or African-Americans. No ethnic difference was noted in calculated measures of insulin resistance such as area under the curve (AUC) for insulin or the insulinogenic index.

A linear regression model was fitted to determine parameters associated with HbA1c, fasting glucose and 2-hour glucose (Table 2a). Fasting glucose, age, female sex and ethnicity were significant predictors of HbA1c (R^2^ = 0.395; F < .001). After adjusting for known confounders, HbA1c was significantly higher among African-Americans and Hispanics than among NHWs. After adjusting for confounders, fasting glucose levels were predicted only weakly by the Homeostasis Model Assessment measure of insulin resistance (HOMA-IR) and by PI use (R^2^ = 0.153; F = .012). However, 2-hour glucose level (adjusted) was strongly and significantly predicted by age, female sex, ethnicity and fasting glucose, (R^2^ = 0.565; F < .001). Hispanics and African-Americans had significantly higher 2-hour glucose levels than NHW’s. In the regression model, for the 2-hour glucose level, the significant interaction effect of ethnicity with fasting glucose (P < 0.001) highlighted differences among the three ethnic groups (NHW’s < African Americans < Hispanics) in this glycemic parameter. Outcomes of the regression model for the glucose response curves in the OGTT are shown in Table 2b. Age, female sex, HOMA-IR and AUC insulin were significant predictors in the model (R^2^ = 0.392; F < 0.001). There was a significant interaction of ethnicity with CD4 strata.

**Table 2:** Multivariate regression models: factors associated with (a) HbA1c, Fasting Glucose, 2-Hour Glucose, and (b) glucose response curves in the OGTT

**Variable**	**Standardized Regression Coefficient**	**P-value**
**HbA1c**		***R***^***2***^ ***= 0.395***
**(a)**		
Fasting Glucose	.533	.011
Female (versus male)	.356	.005
Age	.193	.003
Ethnic Category	301	.038
White	Referent	
African American	.322	.005
Hispanic	.167	.039
**Fasting Glucose**		***R***^***2***^ ***= 0.153***
HOMA-IR	.325	<.001
PI Drug	-.154	.050
**2-Hour Glucose**		***R***^***2***^ ***= 0.565***
Fasting Glucose	.325	.010
Age	.292	.032
Female (versus male)	.360	.003
HOMA-IR	.141	.005
Ethnic Category	.146	<.001
White	Referent group	
African American	-.126	.014
Hispanic	-.380	<.001
Ethnicity*Fasting Glucose	.165	<.001
**(b)**		
**Glucose Response Curves in OGTT**		***R***^***2***^ ***= 0.392***
Age	.276	<.001
Female (versus male)	.106	.051
HOMA-IR	.136	.062
AUC Insulin	.145	.080
Ethnicity *CD4	-172	.023

**Figure 1:**
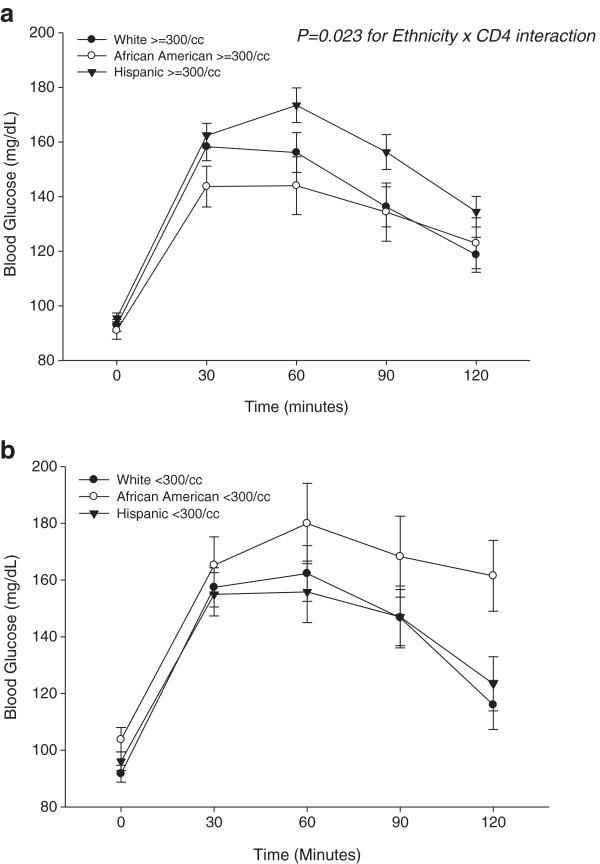
**Glucose response curves for HIV-infected African-Americans, Hispanics and NHW’s in the oral glucose tolerance test.** Multivariate models revealed a significant interaction between ethnicity and CD4+ T cell stratum that affected the glucose response curves (P = 0.023). **a.** Glucose response curves of patients with CD4+ T cell counts ≥ 300/cc on stable HAART; **b.** Glucose response curves of patients with CD4+ T cell counts < 300/cc on stable HAART.

Multivariate analysis (repeated measures ANOVA) assessed the effect of ethnicity on the OGTT glucose response curves after controlling for confounders (age, sex, race, obesity, fasting glucose, HOMA-IR, family history of diabetes, blood pressure, duration of HIV, and PI drug use) and also measured interactions of ethnicity with age, sex and CD4 strata. Ethnicity alone did not distinguish the glucose response curves in a statistically significant manner. Interaction effects of ethnicity*sex, and ethnicity*age were also not significant, but an interaction between ethnicity and CD4 strata was significant for the glucose response curves (P = 0.023). Interaction effects of ethnicity with CD4 count imply that the effect of ethnicity is modified by the CD4 count of the subjects. To elucidate how ethnicity affects the relationship between glycemic excursions and CD4 levels, we plotted post-challenge glucose data against ethnic groups for each CD4 stratum (Figure 1). In the moderate-high CD4 group, Hispanic subjects had the highest mean post-challenge glycemic excursion (Figure 1a), while in the low CD4 group, African-Americans had the highest mean post-challenge glycemic excursion (Figure 1b).

## Discussion

This is the first study to assess ethnic differences in glycemic parameters among HIV patients on a stable HAART regimen, specifically those with hypertriglyceridemia. The selection criteria for the Heart Positive study permitted us to investigate metabolic factors that differentiate glycemic control between ethnic groups of HIV patients without a history of diabetes, and to analyze a large data set of both fasting and post-prandial glucose responses. In bivariate analyses, African-Americans, Hispanics and NHW’s had similar fasting glucose levels, but African-Americans had higher HbA1c and 2-hour OGTT glucose values than Hispanics and NHWs. Significant ethnic differences noted in the multivariate models for 2-hour glucose and HbA1c suggested that ethnicity affects postprandial glycemic control in HIV patients on HAART. These effects persisted after adjusting for group differences in age, sex, HIV years, HAART years, smoking, obesity, fasting glucose, fasting insulin, and lipid levels. Overall, African-Americans and Hispanics had higher glucose values after oral glucose challenge as well as higher HbA1c levels than NHW’s, and an interaction between ethnicity and CD4 count modulated the glycemic response.

A previous study demonstrated that HIV-infected African American women have higher HbA1c levels than NHWs [10]. While controlling for ethnicity, age and CD4 count provided a reasonably accurate reflection of glycemic control in that study, the present results indicate that the greater impairment in glycemic control among HIV infected African-Americans is not due to unmeasured or suboptimally measured risk factors. A robust model that included all possible risk factors (demographic, anthropometric, clinical, and metabolic parameters) improved our confidence in the existence of ethnic disparities in specific glycemic parameters in this cohort who are at high risk for metabolic complications. It is possible that the effect of ethnicity in part “pre-dated” HIV infection or HAART therapy, since African American and Hispanics the general US population is known to have increased risk for impaired glucose tolerance and diabetes compared to NHW’s. The cross-sectional design of this study and absence of a HIV-negative control group did not permit us to estimate the specific effects of HIV / HAART on the risk of dysglycemia. However the interaction between ethnicity and CD4 level suggests that specific effects related to the immune status resulting from chronic HIV infection and its treatment magn ify the risk of dysglycemia in minority ethnic groups.

Previous studies have shown inconsistent associations of CD4 cell count with abnormal glucose levels in HIV patients. Low CD4 cell count was associated with IGT and diabetes in patients co-infected with HIV, hepatitis C and hepatitis B virus [16]. Estrada et al. reported that patients with HIV-associated lipodystrophy and IGT have lower CD4 counts than normoglycemic patients [17]. El-Sadr et al. found an inverse relationship between CD4 counts and insulin resistance in HIV patients [18], but no relationship between CD4 counts and fasting glucose concentrations. This finding is consistent with our data which showed CD4-related differences relate to post-challenge glucose levels, not to fasting glucose levels. Shikuma et al. reported no association between fasting glucose levels and CD4 levels but did not measure post-challenge glucose [19]. In contrast to our results demonstrating no differences in either fasting or post-challenge insulin levels, these investigators also noted that their small sample (7–8 subjects in the respective groups) of HIV patients with CD4 <200/cc had higher fasting insulin levels than those with CD4 >500/cc.

The design of this study makes it difficult to specify the reasons for the relationships between ethnicity, CD4 counts and glucose tolerance in these patients. However, the literature suggests potential mechanisms that should be investigated in future studies. Strictly speaking these results may be generalizable to only to HIV patients with hypertriglyceridemia, however such patients comprise a large proportion of those with HIV infection on various forms of antiretroviral therapy. Additional limitations include the fact that not all ART drug classes or single drugs that could possibly affect insulin sensitivity were included in the multivariate analyses. However, drugs of the protease inhibitor class (which are the most strongly associated with both insulin resistance and defects in glucose uptake) were taken into consideration (i.e., use of PI was controlled for in the analyses).

Abnormal glycemic responses following an oral glucose challenge could be due to insulin resistance or impaired insulin secretion. HOMA-IR, a marker of insulin resistance calculated from fasting values of glucose and insulin, did not differ between patients of the different CD4 strata, suggesting that an aspect of the glycemic defect might involve defective insulin response to an oral challenge. Consistent with this notion, AUC insulin following the oral challenge and the insulinogenic index (a measure of insulin response relative to the glucose surge) did not differ between the two CD4 strata, indicating that persons with both low and high CD4 cell counts may have inadequate insulin secretory response in the face of higher post-challenge glucose levels. It is possible that either low or high CD4 levels might reflect underlying immunologic mechanisms that could affect beta cell mass or function. These possibilities are supported by recent studies showing that, compared to healthy non-HIV persons, HIV patients have higher rates of IGT [20] without necessarily having greater degrees of insulin resistance [21].

While the CD4+ T cell level (low or moderate-high) achieved on a stable HAART regimen was not by itself associated with dysglycemia, ethnicity appeared to interact with low or moderate-high CD4 levels to affect glycemic response to an oral glucose challenge. Activated CD4 cells play a significant role in non-insulin mediated glucose disposal [22]. The association of dysglycemia with low CD4 levels could be due to impaired non-insulin mediated glucose disposal caused by a diminished “sink” of activated CD4 cells (normally equal in total mass to perhaps a third of the liver) or to a greater degree of chronic systemic inflammation. Association of defective glucose disposal with high CD4 counts might be due to post-HAART recovery of CD4 T cells that are unable to utilize glucose effectively because of low GLUT-1 or IL7-R expression or to the influence of genetic factors such as CCL3L1 or CCR5 polymorphisms known to be associated with the development of type 1 diabetes [23]. These findings will be important in formulating hypotheses for an immunologic or molecular basis of impaired glucose metabolism in HIV patients.

These findings have prominent implications for clinical practice. Ethnicity should be considered to affect risk for developing dysglycemia and Metabolic Syndrome among HIV patients. In the U.S., the disproportionately higher burden of HIV/AIDS coupled with the projected population growth among African Americans and Hispanics underscore the need for early screening and intervention as a cost-effective public health strategy [24,25]. Consistent with non-HIV population data [26], we found that African-American ethnicity is associated with IGT, both independently and through an interaction with low CD4 count. Earlier studies investigating the association of ethnicity with IGT or insulin resistance in HIV patients have shown inconsistent results. NHW ethnicity, Hispanic ethnicity, and non-Caucasian race each have been shown to increase risk for insulin resistance or IGT [11]. However, El-Sadr et al. [18] actually found an inverse relationship between African-American ethnicity and insulin resistance in a large HIV cohort, while the Nutrition For Healthy Living study found no relation between ethnicity and insulin resistance in HIV patients [21]. These studies involved predominantly NHW’s [18], whereas our cohort had a mixed ethnic distribution resembling that of the overall US population.

Development of ethnically focused and culturally appropriate treatment approaches is highly relevant to clinical management of the growing HIV epidemic among minorities. The rate of African Americans living with HIV infection (1,202.2) is 8 times higher than that of NHWs (156.7) and 3 times higher than that of Hispanics (487.3) per 100,000 population [24]. HIV’s economic burden is predicted to grow markedly, based on the projected dramatic increase in the minority population as well as in HIV infections and complications associated with HAART [27]. In this context, the present findings in a large, multiethnic cohort of hypertriglyceridemic but otherwise apparently healthy HIV patients on stable HAART have important clinical implications for targeted therapy of HIV/HAART-associated glycemic complications.

## Conclusion

Among hypertriglyceridemic HIV patients on HAART, African-Americans and Hispanics are at increased risk of developing diabetes. Ethnicity also interacts with CD4+ T cell count attained on stable HAART to affect post-challenge glycemic response. Clinicians should understand ethnic-specific factors in developing treatment strategies for dysglycemia, and should be aware of the need for vigilant screening and possibly earlier treatment for HIV-infected African-Americans with low CD4 count and Hispanics with high CD4 count following HAART.

## Abbreviations

(HAART): Highly-active antiretroviral therapy; (BMI): Body mass index; (IGT): Impaired glucose tolerance; (PI’s): Protease inhibitors.

## Competing interests

The authors declare that they have no competing interests.

## Authors’ contributions

RM carried out the data analysis and drafted the manuscript; PC completed the literature review; SR carried out the data analysis and edited the manuscript; DL carried out data analysis and edited the results section; SS finalized the data entry and cleaning; HP completed the laboratory analysis and edited the draft; IC participated in subject recruitment and coordinated the project; DL participated in manuscript writing and edited the drafts; RS edited the manuscript draft; AB supervised the project and drafted/edited the manuscript. All authors read and approved the final manuscript.

## Pre-publication history

The pre-publication history for this paper can be accessed here:

http://www.biomedcentral.com/1472-6823/13/13/prepub

## References

[B1] CDC HIV Prevalence Estimates - United States, 2006 MMWR 2008 57 39 1073 1076 Available at http://www.cdc.gov/mmwr/preview/mmwrhtml/mm5739a2.htm 18830210

[B2] CDC Fact Sheet HIV and AIDS among African Americans 2011 Available at http://www.cdc.gov/nchhstp/newsroom/docs/FastFacts-AA-FINAL508COMP.pdf

[B3] Mondy K Overton ET Grubb J Tong S Seyfried W Powderly W Metabolic syndrome in HIV-infected patients from an urban, midwestern US outpatient population Clin Infect Dis 2007 44 726 734 10.1086/511679 17278068 PMC3170426

[B4] Hadigan C Meigs JB Corcoran C Rietschel P Piecuch S Basgoz N Metabolic abnormalities and cardiovascular disease risk factors in adults with human immunodeficiency virus infection and lipodystrophy Clin Infect Dis 2001 32 130 139 10.1086/317541 11118392

[B5] Vigouroux C Gharakhanian S Salhi Y Nguyen TH Chevenne D Capeau J Diabetes, insulin resistance and dyslipidaemia in lipodystrophic HIV-infected patients on highly active antiretroviral therapy (HAART) Diabetes Metab 1999 25 225 232 10499191

[B6] Kotler DP Thea DM Heo M Allison DB Engelson ES Wang J Relative influences of sex, race, environment, and HIV infection on body composition in adults Am J Clin Nutr 1999 69 432 439 10075327 10.1093/ajcn/69.3.432

[B7] Miech RA Kim J McConnell C Hamman RF A growing disparity in diabetes-related mortality U.S. trends, 1989–2005 Am J Prev Med 2009 36 126 132 10.1016/j.amepre.2008.09.041 19062239 PMC4608016

[B8] Bleyer AJ Hire D Russell GB Xu J Divers J Shihabi Z Ethnic variation in the correlation between random serum glucose concentration and glycated haemoglobin Diabet Med 2009 26 128 133 10.1111/j.1464-5491.2008.02646.x 19236614

[B9] Herman WH Ma Y Uwaifo G Haffner S Kahn SE Horton ES Differences in A1C by race and ethnicity among patients with impaired glucose tolerance in the Diabetes Prevention Program Diabetes Care 2007 30 2453 2457 10.2337/dc06-2003 17536077 PMC2373980

[B10] Glesby MJ Hoover DR Shi Q Danoff A Howard A Tien P Glycated haemoglobin in diabetic women with and without HIV infection: data from the Women's Interagency HIV Study Antivir Ther 2010 15 571 577 10.3851/IMP1557 20587850 PMC2943237

[B11] Howard AA Floris-Moore M Lo Y Arnsten JH Fleischer N Klein RS Abnormal glucose metabolism among older men with or at risk of HIV infection HIV Med 2006 7 389 396 10.1111/j.1468-1293.2006.00398.x 16903984 PMC2104626

[B12] Mehta SH Moore RD Thomas DL Chaisson RE Sulkowski MS The effect of HAART and HCV infection on the development of hyperglycemia among HIV-infected persons J Acquir Immune Defic Syndr 2003 33 577 584 10.1097/00126334-200308150-00005 12902801

[B13] Balasubramanyam A Coraza I Smith EO Scott LW Patel P Iyer D Taylor AA Giordano TP Sekhar RV Clark P Cuevas-Sanchez E Kamble S Ballantyne CM Pownall HM Combination of niacin and fenofibrate with lifestyle changes improves dyslipidemia and hypoadiponectinemia in HIV Patients on ART: results of Heart Positive, a randomized, controlled study J Clin Endocrinol Metab 2011 96 2236 2247 10.1210/jc.2010-3067 21565796 PMC3135191

[B14] Ravussin E Lillioja S Anderson TE Christin L Bogardus C Determinants of 24-hour energy expenditure in man. Methods and results using a respiratory chamber J Clin Invest 1986 78 1568 1578 10.1172/JCI112749 3782471 PMC423919

[B15] WHO WHO HIV/AIDS Do Antiretroviral therapy for HIV infection in adults and adolescents in resource-limited settings: towards universal access 2006 Geneva, Switzerland: World Health Organization

[B16] Gianotti N Visco F Galli L Barda B Piatti P Salpietro S Detecting impaired glucose tolerance or type 2 diabetes mellitus by means of an oral glucose tolerance test in HIV-infected patients HIV Med10.1111/j.1468-1293.2010.00860.x 20629770

[B17] Estrada VM-LM Gonzalez-Lopez A Tellez MJ de Villar N Perez BM Glucose tolerance, insulin, proinsulin and leptin in protease inhibitor-treated HIV-infected patients with lipoatrophy Paper presented at the 13th International AIDS Conference 2000 Durban, South Africa

[B18] El-Sadr WM Mullin CM Carr A Gibert C Rappoport C Visnegarwala F Effects of HIV disease on lipid, glucose and insulin levels: results from a large antiretroviral-naive cohort HIV Med 2005 6 114 121 10.1111/j.1468-1293.2005.00273.x 15807717

[B19] Shikuma CM Waslien C McKeague J Baker N Arakaki M Cui XW Fasting hyperinsulinemia and increased waist-to-hip ratios in non-wasting individuals with AIDS AIDS 1999 13 1359 1365 10.1097/00002030-199907300-00013 10449289

[B20] Grinspoon S Carr A Cardiovascular risk and body-fat abnormalities in HIV-infected adults N Engl J Med 2005 352 48 62 10.1056/NEJMra041811 15635112

[B21] Jones CY Wilson IB Greenberg AS Shevitz A Knox TA Gorbach SL Insulin resistance in HIV-infected men and women in the nutrition for healthy living cohort J Acquir Immune Defic Syndr 2005 40 202 211 10.1097/01.qai.0000165910.89462.2f 16186739

[B22] Jones RG Thompson CB Revving the engine: signal transduction fuels T cell activation Immunity 2007 27 173 178 10.1016/j.immuni.2007.07.008 17723208

[B23] Kalev I Oselin K Parlist P Zilmer M Rajasalu T Podar T CC-chemokine receptor CCR5-del32 mutation as a modifying pathogenetic factor in type I diabetes J Diabetes Complications 2003 17 387 391 10.1016/S1056-8727(02)00242-8 14583186

[B24] CDC Services DoHaH HIV Surveillance by Race/Ethnicity 2008 Atlanta

[B25] Unites States Census Bureau Census Demographic Profiles 2010 Available at http://quickfacts.census.gov/qfd/

[B26] Harris MI Hadden WC Knowler WC Bennett PH Prevalence of diabetes and impaired glucose tolerance and plasma glucose levels in U.S. population aged 20–74 yr Diabetes 1987 36 523 534 10.2337/diabetes.36.4.523 3817306

[B27] Unites States Census Bureau Population Projections Available at http://www.census.gov/population/projections/

